# Accurate measurements of particle emissions from a three-dimensional printer using a chamber test with a mixer-installed sampling system

**DOI:** 10.1038/s41598-023-33538-9

**Published:** 2023-04-20

**Authors:** Handol Lee, Dong-Bin Kwak, Chi Young Choi, Kang-Ho Ahn

**Affiliations:** 1 Department of Environmental Engineering, Inha University, 100 Inha-ro, Michuhol-gu, Incheon, 22212 Republic of Korea; 2 Particle Technology Laboratory, Mechanical Engineering, University of Minnesota, 111 Church St., Minneapolis, S.E. 55455 USA; 3 Department of Mechanical Engineering, Hanyang University, 55 Hanyangdaehak-ro, Sangnok-gu, Ansan, 15588 Republic of Korea

**Keywords:** Engineering, Nanoscience and technology

## Abstract

Recently, three-dimensional (3D) printing has attracted attention as a new manufacturing technology. However, there is lack of data and regulations regarding the emissions of ultrafine particles from 3D printers. Therefore, we investigated particle emissions from a 3D printer using a chamber system. The test system was improved by installing a developed mixer for accurate measurement. Without a mixer, the particle concentration was unstable depending on the sampling point; however, reliable data with good uniformity were obtained by installing a mixer. Using the test system with a mixer, we investigated particle emissions from a 3D printer during operation. Filaments made each of acrylonitrile butadiene styrene (ABS) and polylactic acid (PLA) were used as the printing material. The effects of nozzle temperature and printing time were investigated. Compared to the effect of the printing time, the nozzle temperature had greater impact on the particle emissions. The dominant particle size for the emissions from a 3D printer is less than 10 nm, and the particle concentration decreased with increasing particle size.

## Introduction

Three-dimensional (3D) printers employ an additive manufacturing process in order to layer materials for fabricating 3D structures. Owing to the advantages of 3D printers in both the material costs and simplification of the manufacturing process, 3D printers are increasingly attracting attention both in consumer market and industrial sector^1^. Accordingly, the additive manufacturing is expected to reach 5% of the global manufacturing and achieve 14.5% of the compound annual growth rate by the year 2050^2^. 3D printing technology as a fast-emerging technology has wide scale applications such as in healthcare, agriculture, automotive, and aerospace industries^3–5^. Among 3D printing methods, the fused deposition modeling (FDM) technology is the most popular method. It is based on the extrusion of a thermoplastic filament through a heated nozzle and, subsequently, melting the filament to deposit successive layers for fabricating a 3D object. Although FDM is a promising 3D printing technology in various sectors, e.g., industry, healthcare, and energy, users might overlook the possible negative influences of 3D printers on both environment and human health, which are now becoming serious issues including waste materials, air pollution, and hazardous gaseous and particulate emissions^6–13^. Besides, many consumer-level 3D printers in personal and public settings including homes, universities, schools, and libraries are often not accompanied with a means of filtration units or ventilation systems^14^. Therefore, the studies on protective measures to control 3D printer emissions including ventilation and filtration methods have been also published^15–17^.

During the 3D printing process in FDM, a heated filament undergoes physical and chemical changes, following which particle emissions occur. Extruded filaments emit high concentrations of semi-volatile compounds, which contribute to new particle formation and condensational particle growth^18^. Upon realizing the importance of emission problems, many researchers have recently investigated particle emissions and their effects on indoor environments during the 3D printing process. Kim et al.^19^ employed online and offline analyses to evaluate particulate and gaseous emissions before, during, and after the 3D printing of the filaments made each of acrylonitrile butadiene styrene (ABS) and polylactic acid (PLA), in an exposure chamber. They observed that use of the ABS filament resulted in much higher concentrations of the emitted particles compared to those in the case of the PLA filament. Another interesting study was performed by Yi et al.^20^, examining the effects of both the fabrication of consumables (e.g., hair comb) and device design on the 3D printing emissions. It was revealed that emission characteristics were affected by the material and color of the filament, and even by the design of the 3D structure printed. Furthermore, Stabile et al.^21^ reported the effects of the filament type on the emission characteristics by using ten different filament types, and they subsequently compared the emission rates using the different filament types. Floyd et al.^22^ also characterized the emissions from a 3D printer by adopting an emission factor based on the printed object weight (density), for eight different filaments, and emphasized the risk of potential exposure to high concentrations of particulate emissions for the users of 3D printers. Furthermore, a similar study was performed by Azimi et al.^23^ to estimate the emission behaviors of various filament types. Other than the above-mentioned studies, the effects of the number of nozzles^24^, surface quality of a printed 3D object^25^, and indoor environment during printing activities^26^ on the emission characteristics were also investigated.

All the above-mentioned studies highlighted the importance of emission characterization in the 3D printing process. However, emission rates have not been completely understood, showing that the measured emission rates vary significantly according to experimental setup, test procedure, and sampling location and method^27–31^. Byrley et al.^31^ reported the characteristics of 3D printer emissions including mean size and concentration obtained from the previous publications. Each study used different procedures and methods for sizing and counting particles, resulting in different sets of data. Ding et al.^28^ compared emission characteristics such as particle emission rate and particle diameter between chamber and flow tunnel measurements. In the findings from Ding et al.^28^, the chamber measurements tend to overestimate particle diameter and underestimate the particle emission rate due to particle growth in test chambers. In the study of Kim et al.^19^ regarding filament material, the particle concentration emitted upon printing ABS filaments was approximately 35 times higher than those emitted upon printing PLA filaments. Stephens et al.^32^, however, showed that printing ABS filaments resulted in 10 times higher emission rate than that in the case of PLA filaments. These different results might come from the experimental setup and procedure they employed. Both studies used a chamber method, but different chamber sizes and sampling methods were employed. Kim et al.^19^ used a 1 m^3^ test chamber with sampling cassettes and sampling inlets in the upper part of the test chamber. On the other hand, Stephens et al.^32^ deployed 45 m^3^ furnished and conditioned office space for emission measurement. Poikkimäki et al.^33^ examined nanocluster aerosol emissions of a 3D printer based on filament material and nozzle temperature. They compared their measured ultrafine particle concentrations with several other studies and found that the emission characteristics varied significantly with experimental conditions.

The concentrations of the emissions from a 3D printer or other office equipment fluctuate depending on the operation, and, generally, relatively high concentrations are emitted. Therefore, to accurately measure the concentrations, an appropriate experimental setup is required. Most of the studies on the emissions from a 3D printer use a chamber system, wherein a 3D printer is placed, with an exhaust chamber port where sampling to each instrument occurs. However, insufficient mixing or particle loss and growth inside a test chamber can lead to the misinterpretation of emission rate, and the amount of the supplied clean air inside the chamber might affect the accuracy and uniformity of the particle concentrations when sampling^28,33^. Furthermore, unsteady and fast-changing particle concentrations significantly affect the particle size distribution measurements when using a scanning mobility particle sizer (SMPS), because of the voltage scanning process, which generally takes 1–2 min to obtain one size distribution. Therefore, caution is needed while measuring the size distribution of the emissions.

In this study, we employed an experimental setup equipped with a developed mixer (combination of perforated metals) for accurately measuring the particle emissions from a 3D printer. Using sodium chloride (NaCl) particles, we found the experimental conditions both for the air-exchange rate and design of the mixer to achieve high accuracy and good uniformity of the particle concentration. Thereafter, the experimental setup was used to evaluate the particle emissions from a 3D printer, depending on both the nozzle temperature and printing time. Filaments made of each ABS and PLA, the most widely used materials for a 3D printer, were examined. Experiments were systematically performed in this study, focusing on high accuracy and uniformity of emissions while performing measurements. The entire system including a mixer can be widely used not only for measuring the emissions from a 3D printer, but also for the evaluating the contaminants emitted from various home- and office-based equipment.

## Methods

### Test chamber system

**Figure 1:**
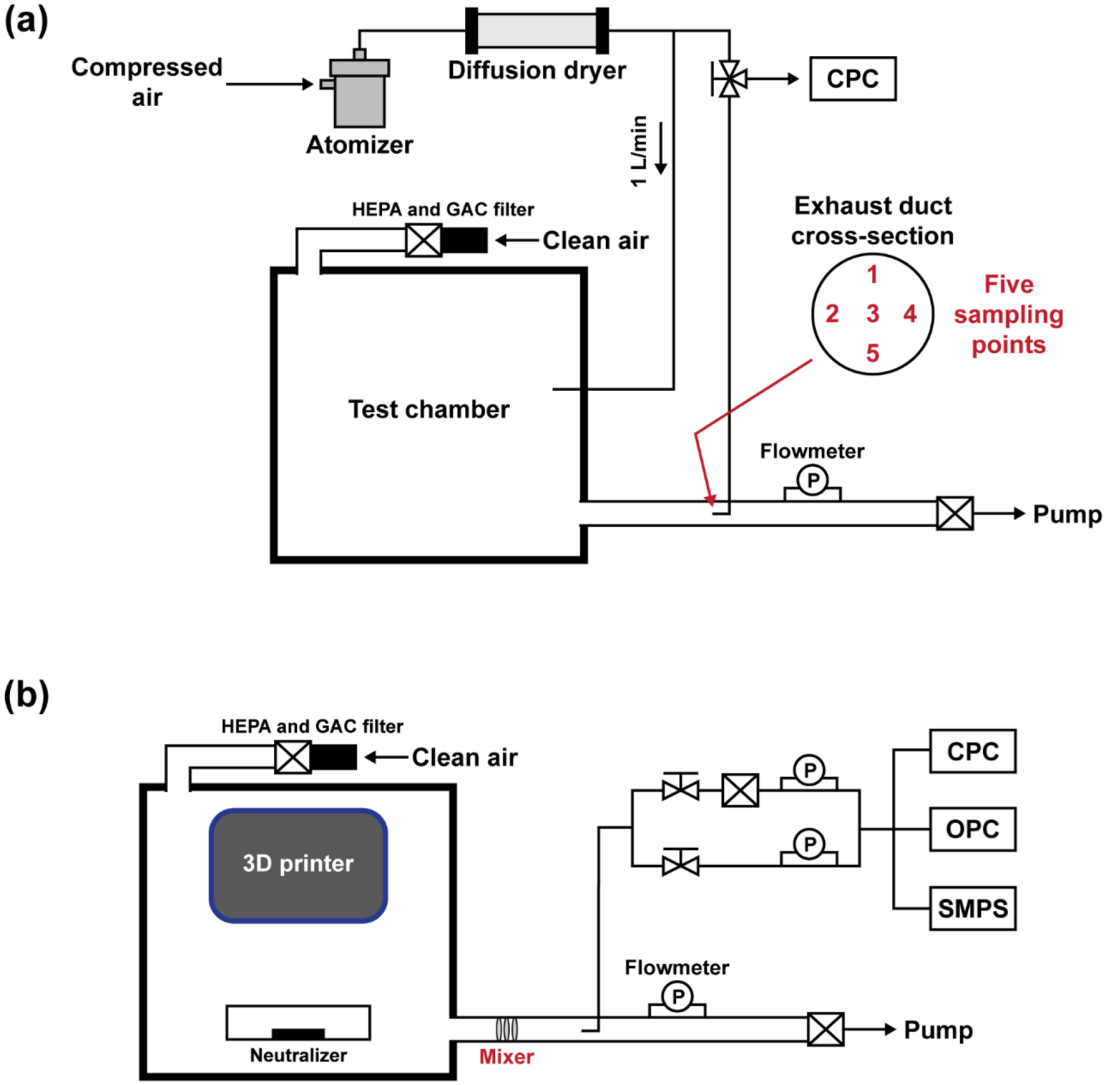
Experimental setups for (**a**) the performance test of mixers and (b) measurements of emissions from a 3D printer.

In this study, we employed a chamber test method for measuring the particle emissions from a 3D printer. The chamber system was first evaluated using NaCl particles as a contamination source in order to ensure the reliable measurement data, by using the setup depicted in Fig. 1a. The 0.5 wt% NaCl solution was aerosolized using a commercial Collison atomizer (model 4810, HCT, Republic of Korea) and dried by a diffusion dryer filled with silica gel to generate NaCl particles. The generated NaCl particles were introduced to the chamber (1 m × 1 m × 1 m), inside which a 3D printer would be placed. Clean air was also supplied to the chamber and mixed with the particles, following which the particle-laden flow was sampled at the downstream of the duct with the diameter of 5 cm connected to the chamber to measure the particle concentration using a condensation particle counter (CPC, model 3775, TSI inc., Shoreview, MN) at the sampling flow rate of 1.5 L min^−1^. The 50% cut-off size of the CPC is approximately 4 nm. The CPC can count particles up to 50,000 # cm^−3^ in a single particle counting mode with continuous, live-time coincidence correction and up to 10^7^ # cm^−3^ in a photometric mode, which has a concentration accuracy of ± 20%. The flow through the duct was controlled using a vacuum pump, which was installed after a filter at the exit. Owing to the relatively large space in the chamber and the chamber exhaust duct, if the particles were not sufficiently mixed or poorly distributed in the duct, inaccurate or unstable concentration data could be obtained depending on the sampling position. Therefore, for ensuring accurate and reliable measurements, a uniform concentration should be ensured at the sampling spot indpendently of the sampling position. To that end, we developed a mixer, which is a combination of perforated metals. The performance of the mixer was evaluated thoroughly by comparing it to the performance of the case in which the mixer was absent. Furthermore, the adequate exchange rate of the clean air in the recirculation system was determined for achieving stable particle concentration measurements. The design and oprating conditions will be discussed later in the section.

Figure 1b depicts the experimental setup for measuring the particle emissions from a 3D printer. The 3D printer was placed and operated inside the chamber. The specifications of the 3D printer are presented in Table 1. The 3D printer is based on FDM and uses both ABS and PLA filaments. The filament diamter is 1.75 mm, and extruder nozzle diameter is 0.4 mm. The layer height of the extruded plastic is 0.1–0.3 mm. The particle emissions generated upon operating the 3D printer were introduced to a CPC, an optical particle counter (OPC, model EDM 180, Grimm Aerosol Technik Company, Germany), and an SMPS. The sampling flow rate of the OPC was 1.2 L min^−1^, the OPC measures particles ranging in size from 0.23 to 20 μm. The SMPS system for measuring size distributions of emissions employed in this study consists of a long differential mobility analyzer (model 4210, HCT, Republic of Korea), high-voltage power supply (model 205B, Bertan, US), and flow system including pumps and mass flow controllers. Size distributions of particles in the size range from 7 to 300 nm were obtained using the SMPS. The TSI Aerosol Instrument Manager software was used for inversion of measured CPC number concetnrations to size distribution, considering particle diffusion losses in the system. Notably, we calibrated all flow systems prior to every experiment, and the particle counters used in this study had been annually calibrated as recommended by manufacturers. The dilution ratio of the particle emissions to the filtered clean air was set to be 1:6 in order to protect the instruments from the high concentration of the emissions. Furthermore, a soft X-ray neutralizer (model SXN-05U, Sunje, Republic of Korea) was placed inside the chamber to minimize the particle losses due to electrostatic effects.

**Table 1:** Specifications of the tested 3D printer.

Main body size (W × D × H) [mm]	306 × 420 × 357
Weight [kg]	13
Material of main body heat bed	Anodized aluminum
Filament diameter [mm]	1.75
Nozzle diameter [mm]	0.4
Layer thickness [mm]	0.1–0.3

### Mixer design

**Figure 2:**
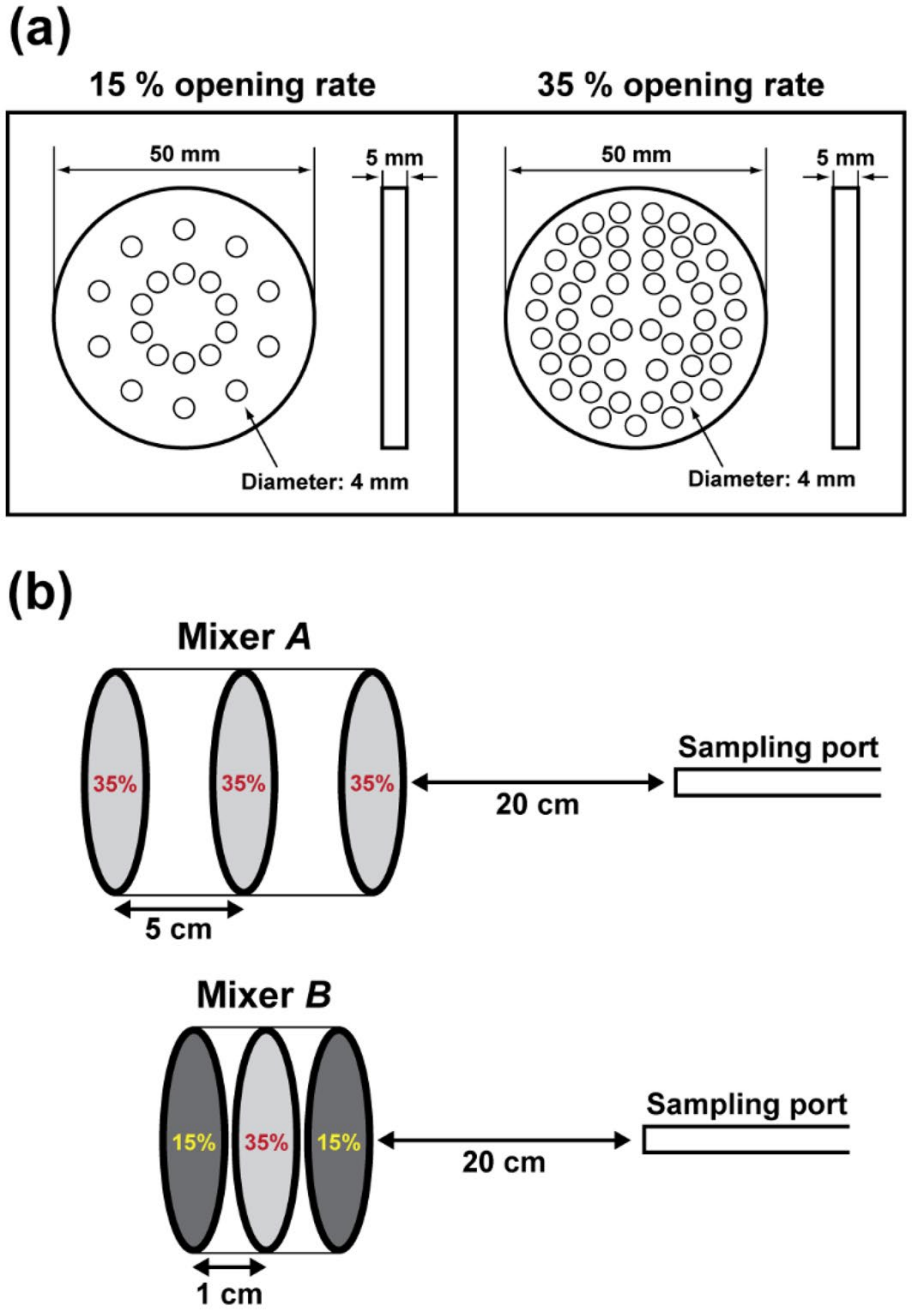
Information of Mixer *A* and*B*: (**a**) design of two perforated metals with 15% (20 holes) and 35% (51 holes) opening rates; (**b**) two combinations of the perforated metals.

In this study, we developed a mixer for achieving the stable sampling of the particle emissions. It was installed at the exit of the chamber before the sampling probe, as depicted in Fig. 1b. Furthermore, we designed two types of perforated metals at different opening rates of 15% (20 circular holes each of diameter 4 mm) and 35% (51 circular holes each of diameter 4 mm), as depicted in Fig. 2a, and the mixers were fabricated through precision processing. Two mixer designs each resulting from two different combinations of the perforated metals are depicted in Fig. 2b. In the first mixer design, three perforated metals, each having 35% opening rate, are placed with a 5-cm spacing between each of them; this mixer will be denoted by Mixer *A*. In the second design, which will be denoted by Mixer *B*, a perforated metal having 15% opening rate was first placed at front, followed by two perforated metals, each having the opening rates of 35% and 15%, respectively, with the constant spacing of 1 cm between each of them, as depicted in Fig. 2b. The sampling was performed 20 cm away from the last perforated metal.

### Test procedure for mixer evaluation

To evaluate the mixer performance regarding the accurate measurements of particle concentration, the test results estimated using each of Mixers *A* and *B* were compared to the data obtained without a mixer. Furthermore, the test results were compared with the theoretically obtained concentrations based on the flow conditions, e.g., dilution ratio and exchange rate in the chamber, and generated upstream NaCl concentration. The exchange rates of the clean air were set to be 1, 2, 3, and 4 h^–1^, and the corresponding clean air flow rates supplied to the chamber were 15.7, 32.3, 49.0, and 65.7 L min^–1^, respectively. Therefore, the theoretical concentration can be obtained by the measured upstream NaCl concentration multiplied by the dilution ratio inside the chamber, e.g., 1/(15.7 + 1) for the 1 h^−1^ exchange rate. To obtain the flow rates of clean air introduced to the chamber, the flow rate of the pump (shown in Fig. 1a) was set to be 15.2, 31.8, 48.5, and 65.2 L min^−1^ for the exchange rate of 1, 2, 3, and 4 h^–1^, respectively, by considering the flow rate of the CPC, which is 1.5 L min^−1^. The concentrations measured at five sampling locations (1, 2, 3, 4, and 5 in Fig. 1a) were compared with one another to evaluate the uniformity of the concentration at the sampling location.

### Test procedure for emission measurement of 3D printer

**Figure 3:**
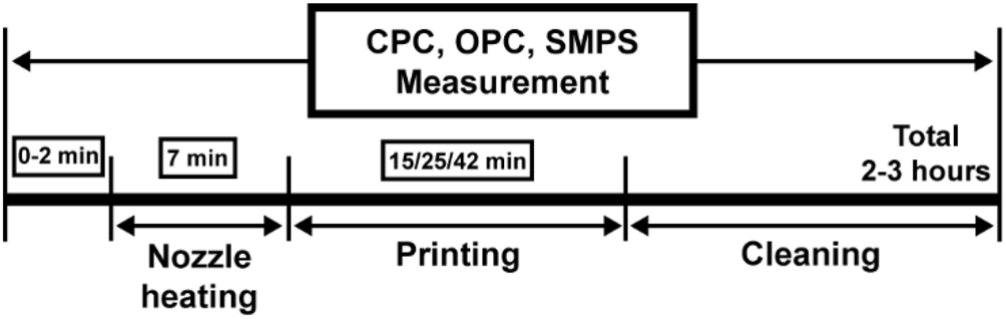
Schedule of an emission test including nozzle heating, printing, and cleaning processes.

For the 3D printer emission test, each test was conducted for three hours including preheating, printing, and cleaning processes. The overall procedure is depicted in Fig. 3. After initiating the measurements, the nozzle of the 3D printer was preheated for seven minutes, following which a 3D structure was printed. For evaluating the effect of the nozzle temperature on the emissions, an 8 mm × 8 mm × 8 mm sized cube was printed for 10 min using the ABS and PLA filaments, and their initial nozzle temperatures were set to be 230–250 °C and 200–220 °C, respectively. This is because the recommended operating temperatures of the ABS and PLA filaments were 240 °C and 210 °C, respectively. We monitored the nozzle temperatures accurately by using a thermocouple attached to the nozzle, and the temperature data were recorded using a data-acquisition software (Labview, National Instruments).

The particle emission characteristics were investigated according to the printing time. In this case, a cube of size 10 mm × 10 mm × 10 mm was fabricated in the printing time of 15, 25, and 42 min. Both the ABS and PLA filaments were used, and their nozzle temperatures were set to be 240 °C and 210 °C, respectively. Furthermore, the total number concentrations of small and large particles emitted from the 3D printer were measured using a CPC and OPC, respectively, and the particle size distribution was also obtained using an SMPS system.

## Results and discussion

### Performance of mixers

#### Without a mixer

**Figure 4:**
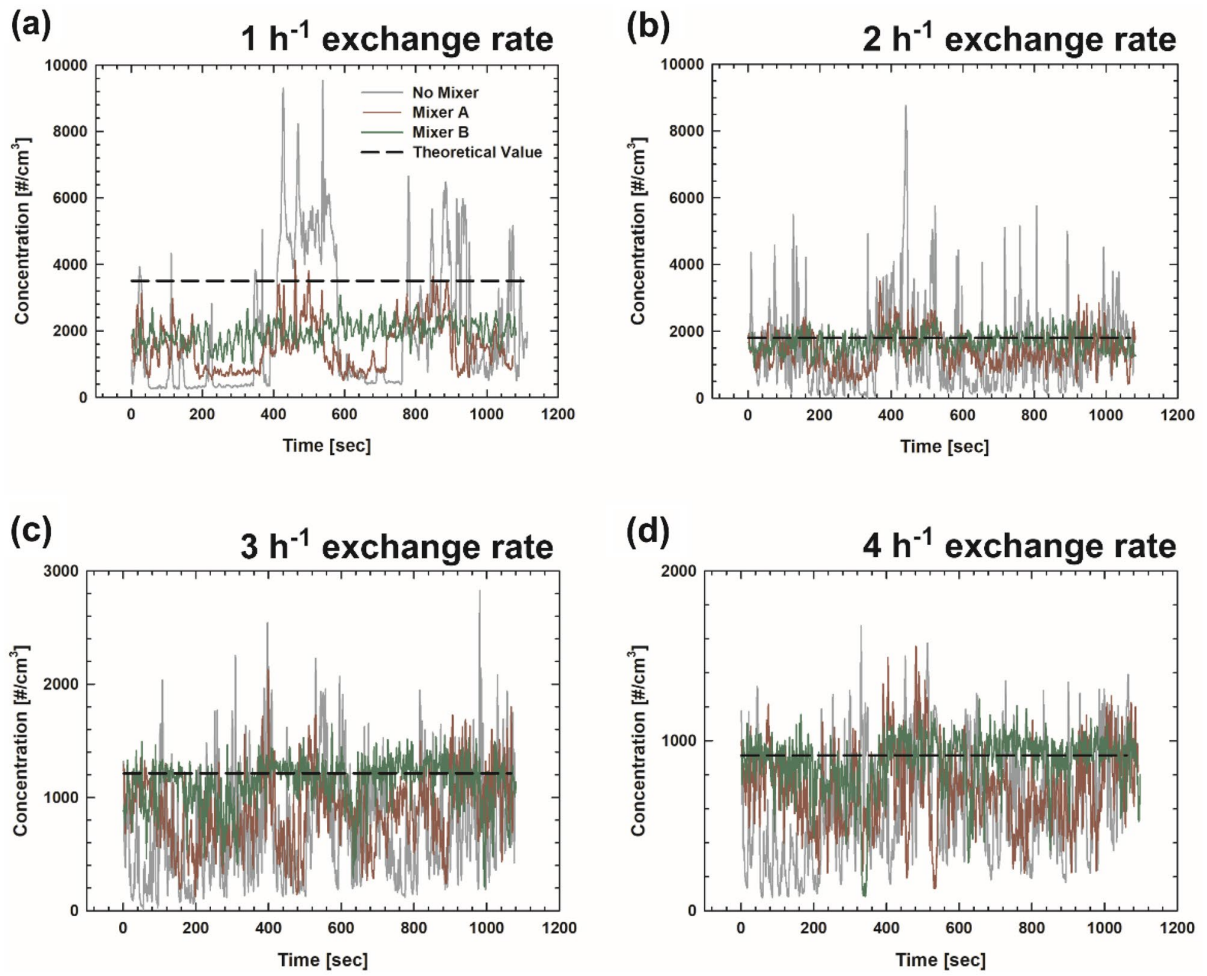
Concentration as a function of time measured at the center of the sampling location (sampling point 3 in Fig. 1a) with and without a mixer for the exchange rate of (**a**) 1 h^−1^, (**b**) 2 h^−1^, (**c**) 3 h^−1^, and (**d**) 4 h^−1^.

**Figure 5:**
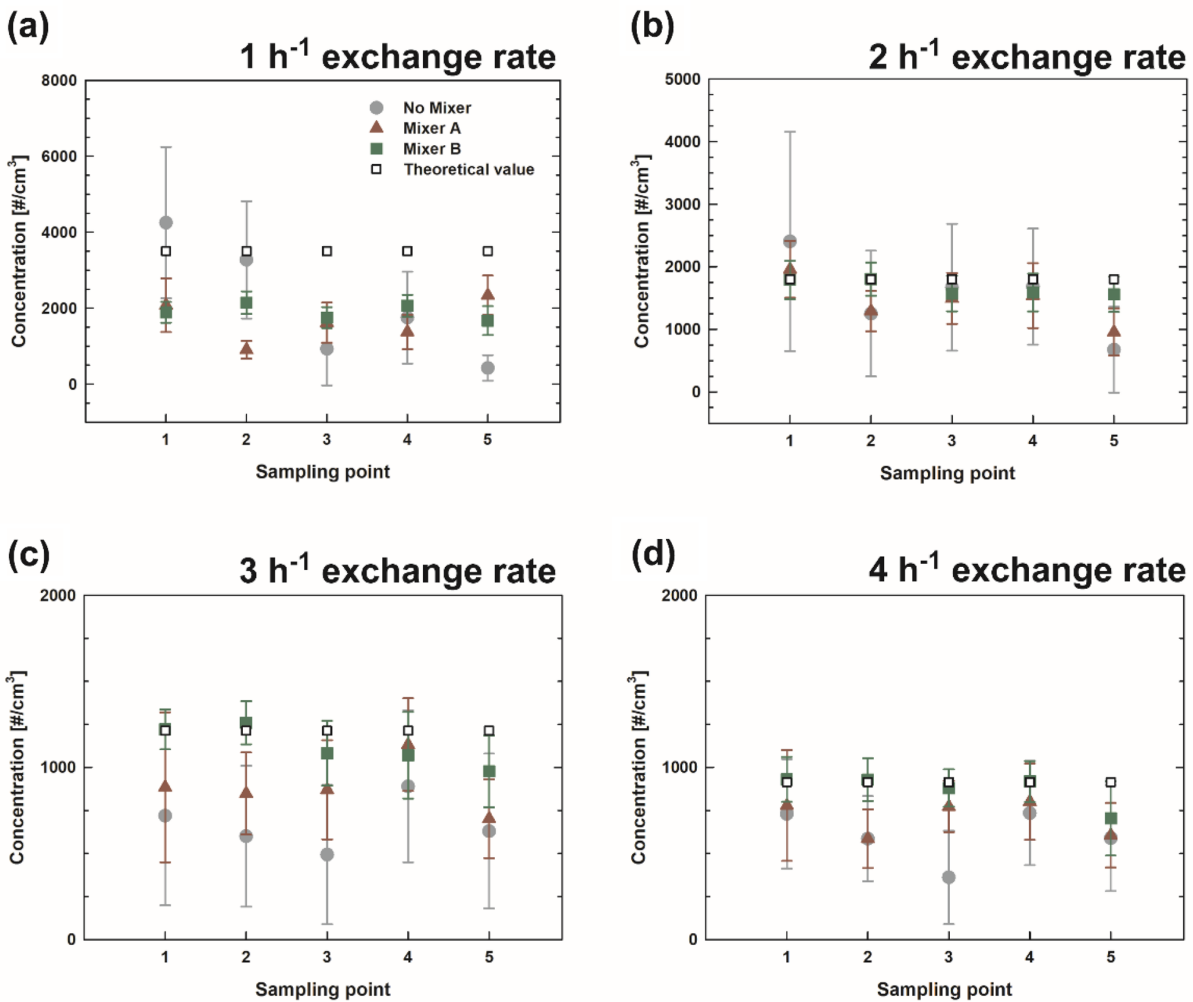
Concentration measured at different sampling points with and without a mixer for the exchange rate of (**a**) 1 h^−1^, (**b**) 2 h^−1^, (**c**) 3 h^−1^, and (**d**) 4 h^−1^.

Prior to evaluating the developed mixers, NaCl particles were used as contaminants without a 3D printer and a mixer. Figure 4 depicts the total number concentrations measured at the center of the exhaust duct, i.e., sampling point 3 in Fig. 1a, with and without a mixer as a function of time. The measurement results for different exchange rates are presented separately, and the theoretical values are denoted as a black dashed line. Figure 5 depicts the particle concentration obtained with and without a mixer at each sampling point. The data point and error bar indicate the average concentration and standard deviation, respectively, and open square symbols represent the theoretical values according to exchange rate. The concentration represents the average of the concentrations measured for approximately 18 min. The reliability of the measurement system can be assessed using two essential points: (1) the ratio of the measured concentration to the theoretical value (accuracy) and (2) the difference between the data obtained at different sampling points (uniformity). We obtained the averaged values of the concentrations measured at five sampling points (gray circular, red triangular, and green square symbols in Fig. 5) for each exchange rate, following which they were normalized using the theoretical value (open square symbol in Fig. 5). Subsequently, their normalized averages and standard deviations were presented in Table 2. Without a mixer, the highest ratio of 79% was obtained at the exchange rate of 2 h^–1^, and other cases without a mixer underestimated the concentrations significantly. At higher exchange rates (3 and 4 h^–1^), the uniformity of the concentration was improved, showing smaller variation in concentration. The variation is the consequence of insufficient mixing in the chamber, resulting in unstable concentrations during the sampling.

**Table 2:** Average and standard deviation of concentrations measured at five sampling points normalized by the theoretical concentration (without a mixer and with Mixers *A* and *B*).

	Exchange rate [h^−1^]			
	1	2	3	4
Without mixer	0.54 ± 0.41	0.79 ± 0.33	0.58 ± 0.13	0.66 ± 0.14
Mixer *A*	0.43 ± 0.16	0.79 ± 0.17	0.72 ± 0.11	0.78 ± 0.09
Mixer *B*	0.56 ± 0.05	0.92 ± 0.06	0.92 ± 0.08	0.95 ± 0.09

#### With Mixers *A* and *b*

Figures 4 and 5 also depict the concentrations measured when installing Mixer *A* (35–35–35% with a 5-cm spacing) and Mixer *B* (15–35–15% with a 1-cm spacing). For the case of Mixer *A* at the exchange rate of 1 h^−1^ in Fig. 4a (red line), the concentration trend according to exchange rate seems to be similar to that for the case without a mixer (gray line), showing pronounced fluctuation during the measurement at the center of the outflow duct. For Mixer *A* as shown in Fig. 5 and Table 2, the lowest concentration ratio of approximately 0.42 was obtained between the measured and theoretical values at the exchange rate of 1 h^–1^, and ratios more than 0.7 were obtained at the other exchange rates. Furthermore, the standard deviations of the measured concentrations at five sampling points were reduced to approximately 0.1 for the exchange rates of 3 and 4 h^−1^ compared with those for the cases of lower exchange rates. Therefore, Mixer *A* improves the performance of the evaluation system in terms of accuracy and uniformity compared with the case without a mixer.

For the case of Mixer *B* at the exchange rate of 1 h^−1^ in Fig. 4a (green line), the measured concentration, as a function of time, did not vary significantly compared to the previous cases (i.e., without a mixer and with Mixer *A*). However, underestimation still exists at this exchange rate, i.e., ratio of 0.56 as shown in Table 2. The ratio might be significantly affected by the exchange rate, especially when it is too low. This might be a consequence of the diffusion loss in the system owing to the lower flow rate. For the cases of Mixer *B* at all exchange rates in Fig. 5 (green square symbols), the variation among the values at different sampling points was considerably reduced, showing that the standard deviations were generally lower than those in the cases without a mixer and with Mixer *A*. Importantly, the ratios of the measured concentrations to the theoretical ones at the exchange rates of 2, 3, and 4 h^–1^ were estimated to be more than 0.9. These findings indicate that by using Mixer *B* at a sufficiently high exchange rate, one can obtain stable and reliable outputs from the testing system. Therefore, we analyzed the characteristics of the particle emissions from the 3D printer by employing Mixer *B* at the exchange rate of 2 h^–1^, and the corresponding flow rate of the pump in Fig. 1b is 29.6 L min^−1^. Notably, in many research works based on the assessment of indoor pollutants emitted by office equipment, the emissions were measured without being mixed sufficiently or with a small sampling probe close to the emission source^6,7,20,34,35^. The data obtained from these evaluation systems might not represent all the particles emitted from various sources in the equipment. However, employing our measurement system enables the measurement of total emissions generated from all the sources.

### Emission from a 3D printer

#### Total concentration

**Figure 6:**
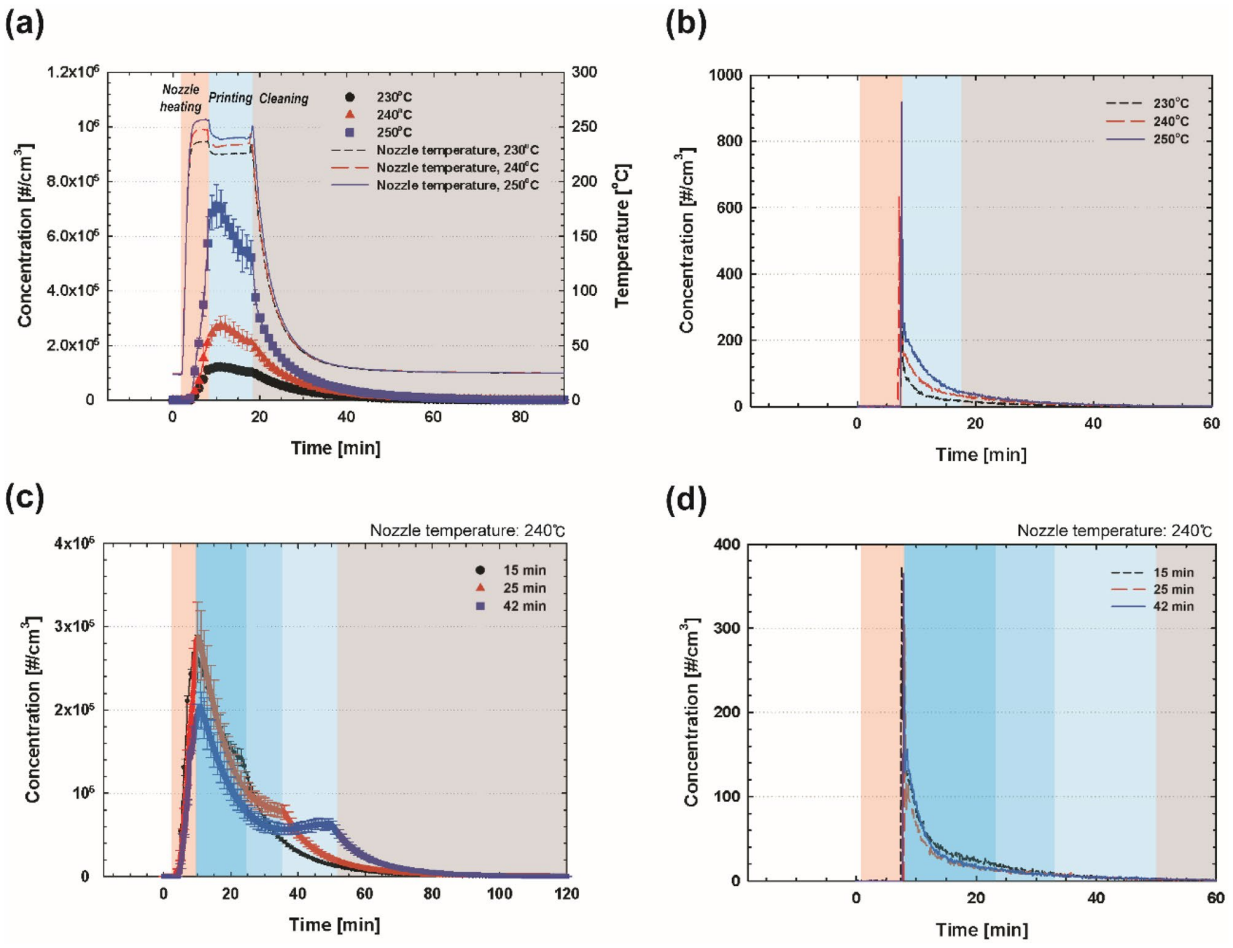
Concentrations of emissions from the ABS filament for different (**a**, **b**) nozzle temperatures and (**c**, **d**) printing times. (**a**) and (**c**) were obtained using a CPC, and (**b**) and (**d**) were obtained using an OPC. The red, blue, and gray shaded areas represent the process of nozzle heating, printing, and cleaning, respectively.

**Figure 7:**
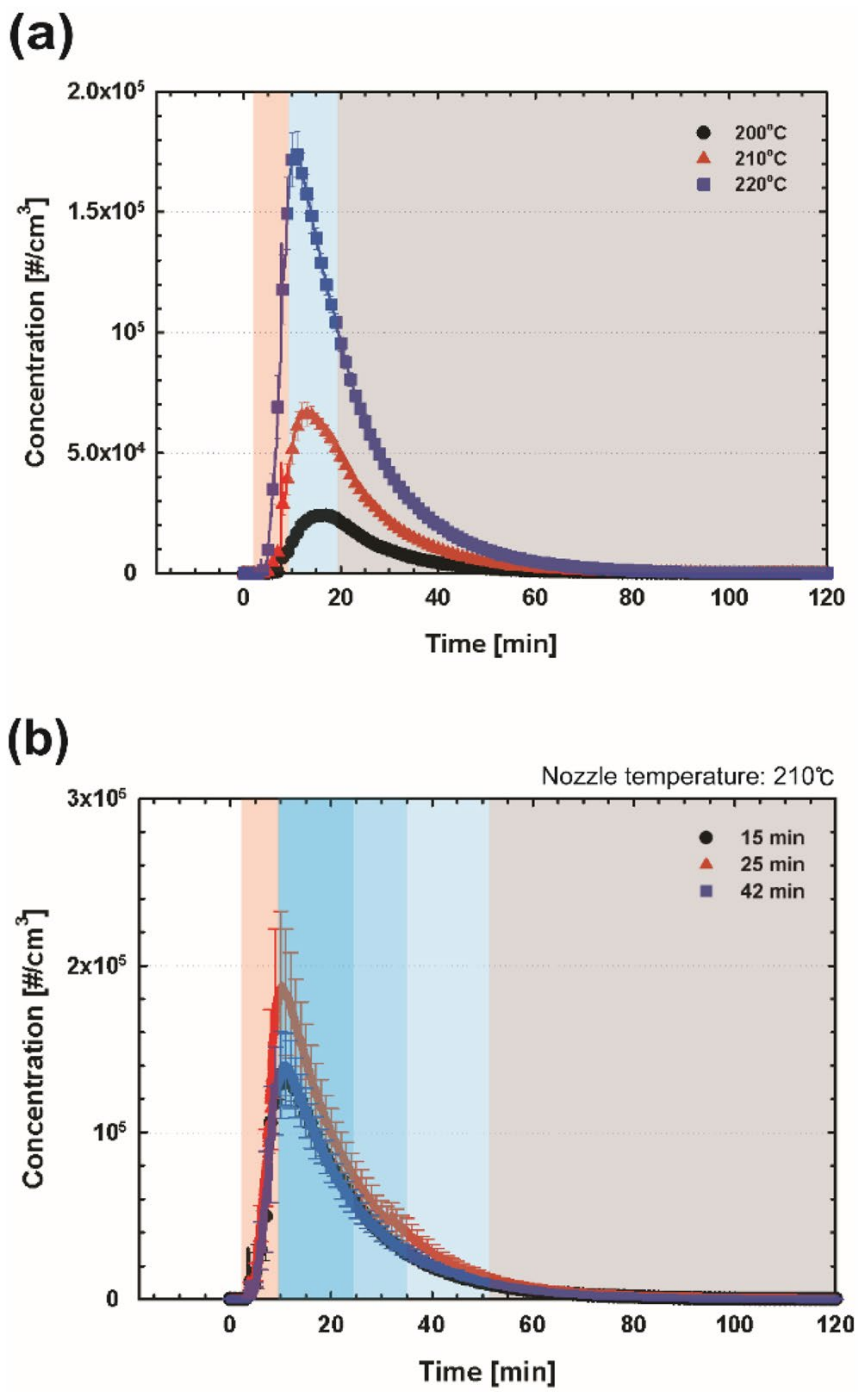
Concentrations of emissions from the PLA filament for different (**a**) nozzle temperatures and (**b**) printing times measured using a CPC. The red, blue, and gray shaded areas represent the process of nozzle heating, printing, and cleaning, respectively.

Figure 6 depicts the ABS emission data for small and large particles generated during the whole process, at different temperature settings and printing times. The red, blue, and gray shaded areas in Figs. 6 and 7 represent the process of heating nozzle, printing, and cleaning, respectively. Figure 6a,b depict the total concentration data obtained using a CPC and OPC, respectively; generally, higher concentration was obtained for higher nozzle temperatures. Interestingly, only a small temperature variation of 10 °C at the nozzle significantly affected the particle concentration for ultrafine particles, i.e., more than doubled peak concentration. During the nozzle-heating process, the particle concentration increased constantly. When the nozzle temperature reached near the set point, the particle concentration reached the highest level. Since the beginning of the printing process, the concentration started to decrease as the nozzle temperature dropped and maintained a certain level while printing a 3D structure. Toward the end of the printing process, the concentration decreased exponentially with time. The interesting findings are depicted in Fig. 6b, showing that the particles of size greater than 0.23 µm were not generated during the preheating process, but that the concentration soared at the beginning of printing. Subsequently, it decreased exponentially with time. However, for these larger particles (> 0.23 µm), the total number concentration is almost negligible compared to that of the smaller particles.

The effects of the printing time on the particle emissions were investigated for the ABS filament at the nozzle temperature of 240 °C, and the total concentrations measured using the CPC and OPC are depicted in Fig. 6c,d, respectively. Compared to the 10-min printing time depicted in Fig. 6a, the operation of the 3D printer for a longer printing time apparently revealed the second peak of particle emissions (or a sudden change in the particle concentration), which appeared at the end of the printing process. From the results, it was observed that decreasing-concentration events occurred during the printing process, along with the reduction in the nozzle temperature. For larger particles (> 0.23 µm), the printing time did not significantly affect the emission concentration. As previously seen in Fig. 6b, most of the larger particles are emitted at the beginning of the printing process; therefore, we assume that the printing time has little association with the emissions of large particles.

For the 3D printing using the PLA filament, the trend of the emitted particle concentration was similar to that of the ABS filament, showing the highest total concentration at the highest nozzle temperature in Fig. 7a. However, compared to the ABS filament case, the PLA filament case does not have a distinct boundary (or a sudden change in the particle concentration) toward the end of the printing process. This might result from the relatively low particle emissions from the PLA filament. In Fig. 7b, with regard to the printing time at the nozzle temperature of 210 °C, the highest concentration was obtained for the printing time of 25 min. This trend was also observed when comparing the first peak concentrations for the ABS filament case in Fig. 6c. The contributing factor to this trend should be studied further; however, the results clearly show that the emissions are dominantly generated during the preheating process and the initial stage of the printing process; therefore, longer printing time does not ensure a higher concentration of particle emissions from the 3D printer, indicating less effect of printing time on small particle emissions as well as larger particles. Notably, larger particles (> 0.23 μm) could not be detected by the OPC measurement. Furthermore, the total concentrations obtained under different conditions are summarized in Table 3. Generally, compared with the ABS filament case, differences were observed both in the total concentration and the number of peaks: the overall concentration for the PLA filament case was estimated to be much lower than that in the 3D printing using the ABS filament; the emissions from the operation using the PLA filament exhibited only one peak during the entire cycle that included preheating, printing, and cleaning processes.

**Table 3:** Summary of total particle number for different nozzle temperatures and printing times for ABS and PLA filaments.

ABS	Nozzle temperature [°C]			Printing time [min]		
	230	240	250	15	25	42
Total particle number [#]						
CPC	3.81 × 10^9^	7.46 × 10^9^	1.62 × 10^10^	7.67 × 10^9^	7.97 × 10^9^	7.19 × 10^9^
OPC	7.99 × 10^5^	1.52 × 10^6^	2.10 × 10^6^	1.27 × 10^6^	8.75 × 10^5^	1.08 × 10^6^

PLA	Nozzle temperature [°C]			Printing time [min]		
	210	220	230	15	25	42
Total particle number [#]						
CPC	7.67 × 10^8^	1.99 × 10^9^	4.63 × 10^9^	3.97 × 10^9^	5.31 × 10^9^	4.01 × 10^9^
OPC	–	–	–	–	–	–

#### Particle size distribution

**Figure 8:**
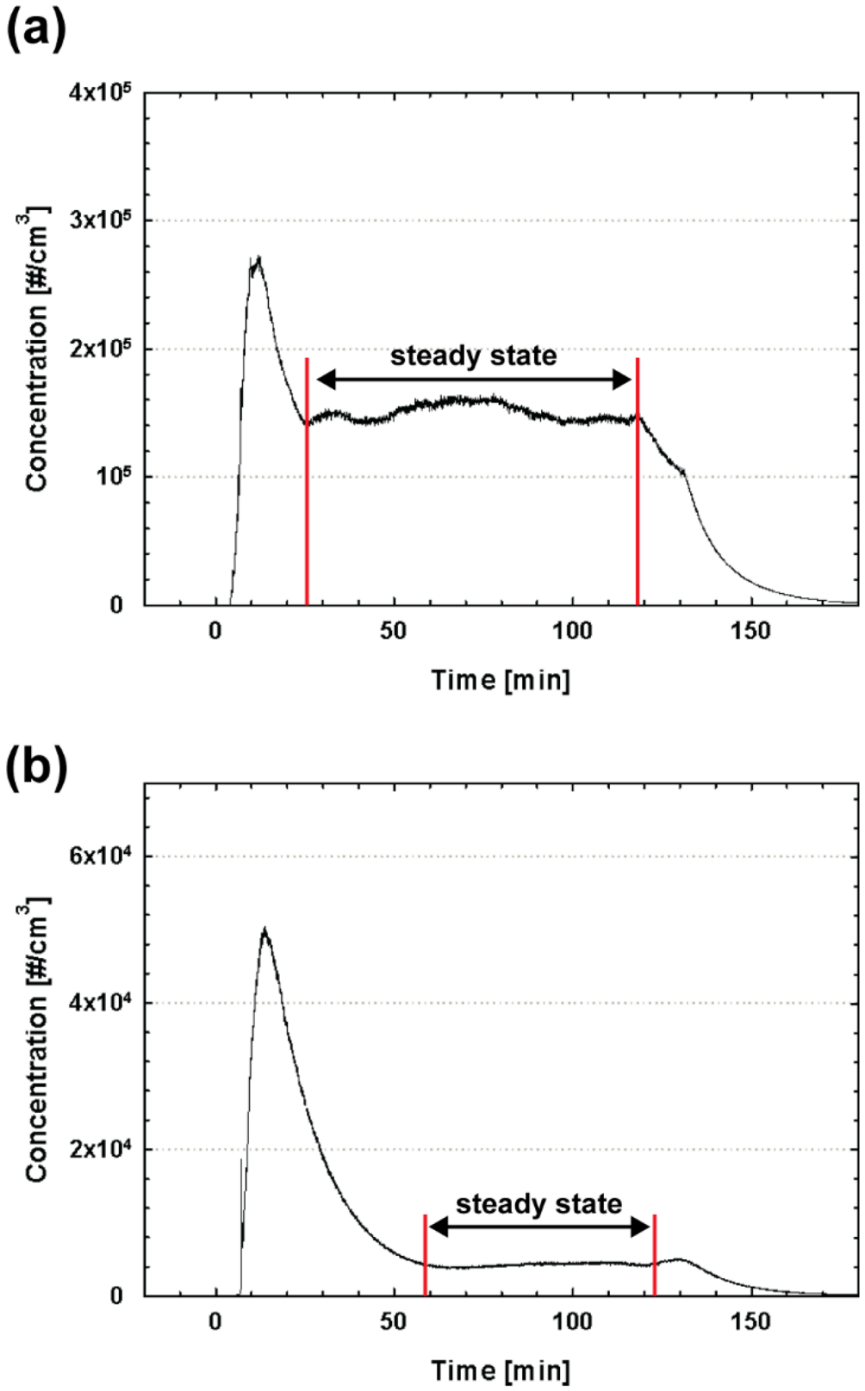
Concentration as a function of time measured using a CPC for (**a**) ABS and (**b**) PLA filaments during two hours of a printing process.

We measured the particle size distributions of the emissions from the 3D printer during a printing process. The scanning time was set to be 90 s, and the particles of size ranging approximately from 7 to 300 nm were scanned. Notably, to measure an accurate particle size distribution using an SMPS, the measurement should be performed at steady-state particle concentration. Therefore, we printed nine cubes with the size of 10 mm × 10 mm × 10 mm to extend the printing time to approximately two hours. The particle concentrations emitted during the entire printing process both for ABS and PLA filaments are depicted in Fig. 8a,b, respectively. For the 3D printing using the ABS filament, the steady-state condition was achieved after 25 min of the experiment, and for the PLA filament case, the steady-state concentration was achieved after 60 min. As discussed in the previous sections, much higher concentration was measured for the ABS filament case compared with the PLA filament case. Notably, for larger particles (> 0.23 μm), we also tried to achieve the steady-state region and obtain the particle size distribution using the OPC; however, during the long printing time of two hours, larger particles were not generated.

**Figure 9:**
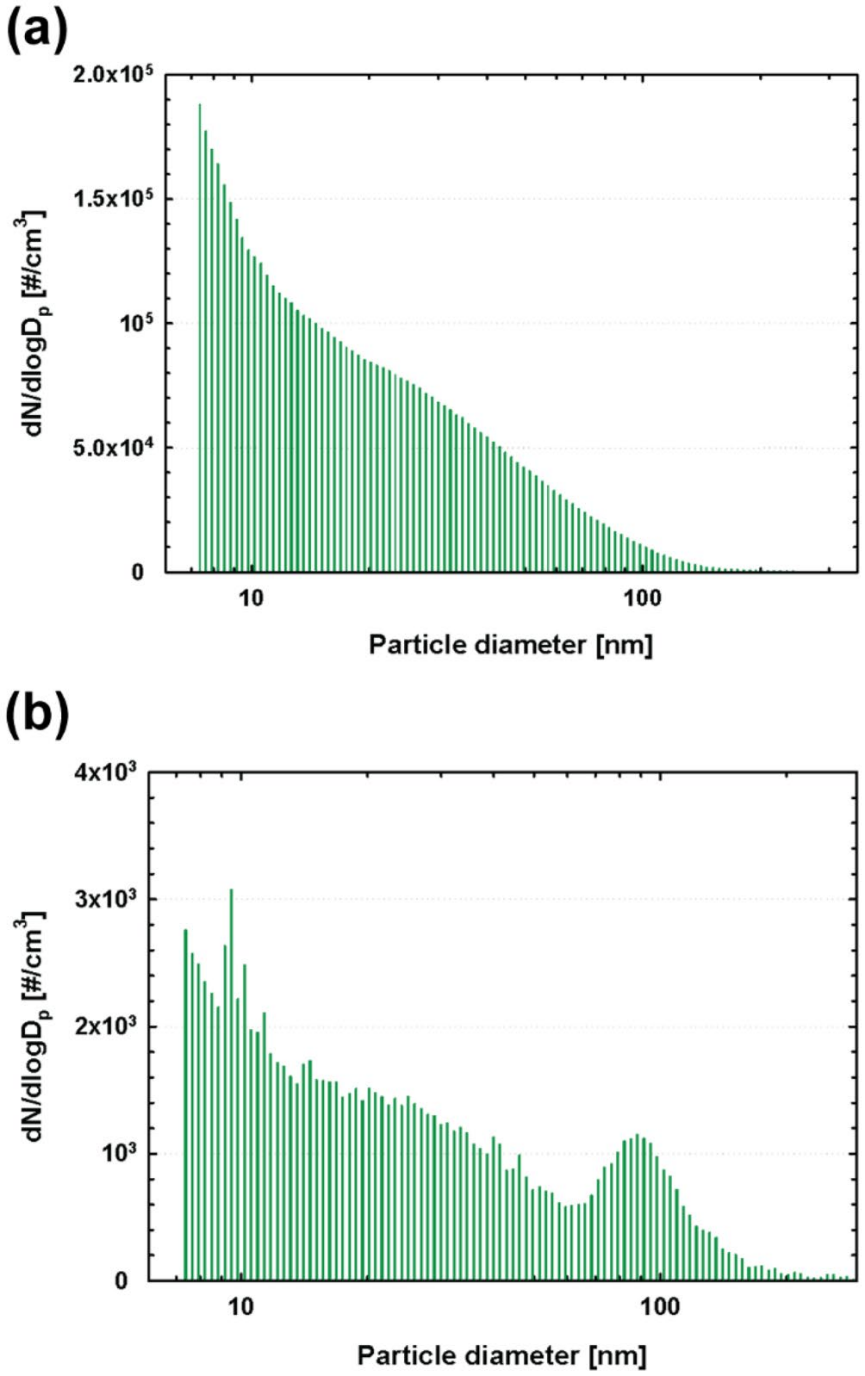
Particle size distribution measured at the steady-state particle concentration region in Fig. 8 for (**a**) ABS and (**b**) PLA filaments.

Figure 9 depicts the particle-size distribution data obtained using the SMPS measurements both for ABS and PLA filaments separately while maintaining the steady-state particle concentrations. The SMPS measurement were continuously performed at the entire steady-state region, and the data obtained were averaged. For the ABS filament case in Fig. 9a, the particle concetration decreased with the decreasing particle size, and thus we can assume that particles smaller than 7 nm are emitted more than the larger particles. The similar trend was observed for the PLA filament case in Fig. 9b, showing that the particle concentration decreases with increasing particle size, and it increases again at approximately 60 nm, with the second peak for the particle size of 90 nm.

## Conclusion

Particle emissions from a 3D printer were analyzed. The emission analysis using an office equipment of relatively large volume like a 3D printer requires a large chamber for placing the equipment inside and an outflow line for sampling particles. Using this setup, it was observed that the uniformity of the particle concentrations, which was a result of sufficient mixing, affected the accuracy of the measurements. Therefore, we developed a mixer using perforated metals to improve the uniformity of the particle concentrations, installed it before the sampling probe, following which we evaluated the performance of the mixer by comparing the measurement data obtained without a mixer. We observed that the mixer design of 15%–35%–15% with a 1-cm spacing (Mixer *B*) showed the highest ratio of the measured particle concentration to the theoretical one under the exchange rates of 2, 3, and 4 h^–1^. Furthermore, using the mixer, the variations among the particle concentrations measured at different sampling points in the sampling plane were minimized. The results of the concentration ratios and variations show that Mixer *B* provides high accuracy and good uniformity of the measurement data. Therefore, we employed this mixer in an experimental setup for characterizing the particle emissions from a 3D printer. We used both ABS and PLA filaments as a printing material, and, subsequently, the effects of both the nozzle temperature and printing time on the particle emissions were evaluated using CPC, OPC, and SMPS measurements. Based on the systematic experiments, the following conclusions were drawn.

1. The nozzle temperature is the dominant variable responsible for the occurrence of ultrafine particles emitted from a 3D printer; however, printing time also exerts a relatively less influence on the total particle emissions.
2. The characteristics of the particle emission vary significantly depending on the filament material, e.g., ABS and PLA. Furthermore, relatively large particles (> 0.23 μm) measured using an OPC were emitted only at the beginning of printing process using the ABS filament; however, these large particles were not generated during the printing process using the PLA filament. Toward the end of the 3D printing process using the ABS filament, the slope of the decreasing particle concentration became steeper; however, the slope of the concentration toward the end of the printing process did not change significantly for the PLA filament case, indicating less influence of the printing time on the particle emissions.
3. Using the SMPS analysis, for both ABS and PLA filaments, the highest concentrations were observed for the particle size smaller than 10 nm, and generally the emissions decreased with increasing particle size.

Notably, the data obtained in this study revealed that sub-10 nm particles emitted significantly from a 3D printer. In this study, we measured total concentrations and size distributions of 3D printer emissions using the CPC that has a 50% cut-off size of 4 nm and SMPS in the size range between 7 and 300 nm. For the better characterization of emissions from a 3D printer, further study should be conducted using instruments for sub-3 nm ultrafine particle measurements. Moreover, the performance of the mixers tested in this study was evaluated by using NaCl particles with a mean particle size of approximately 40 nm, which is larger than that of the real 3D printer emissions, i.e., sub-10 nm. Therefore, the sampling system with the mixers introduced in this study should be further verified using ultrafine particles, simulating the real emissions, and estimating the diffusional loss in the system. Our experimental setup and procedure for the accurate characterization of particle emissions, and the findings from the experiments are expected to be informative for future studies.

## Data Availability

Some or all data, models, or code that support the findings of this study are available from the corresponding author upon reasonable request.
